# NPAS2 promotes cell survival of hepatocellular carcinoma by transactivating CDC25A

**DOI:** 10.1038/cddis.2017.131

**Published:** 2017-03-23

**Authors:** Peng Yuan, Jibin Li, Feng Zhou, Qichao Huang, Jiansheng Zhang, Xu Guo, Zhuomin Lyu, Hongxin Zhang, Jinliang Xing

**Affiliations:** 1 Department of Pain Treatment, Tangdu Hospital, The Fourth Military Medical University, Xi'an, Shaanxi, China; 2 State Key Laboratory of Cancer Biology and Experimental Teaching Center of Basic Medicine, The Fourth Military Medical University, Xi'an, Shaanxi, China; 3 Department of General Surgery, Affiliated Huaihai Hospital of Xuzhou Medical College, Xuzhou, Jiangsu, China

## Abstract

Emerging evidences show that disruption of the circadian rhythm is associated with tumor initiation and progression. Neuronal PAS domain protein 2 (NPAS2), one of the core circadian molecules, has been proved to be a potential prognostic biomarker in colorectal and breast cancers. However, to date, the potential functional roles and molecular mechanisms by which NPAS2 affects cancer cell survival are greatly unclear, especially in hepatocellular carcinoma (HCC). We first investigated the expression of NPAS2 and its clinical significance in HCC. We then systematically explored the role of NPAS2 in HCC cell survival both *in vitro* and *in vivo* and the underlying mechanism. NPAS2 was frequently upregulated in HCC, which significantly facilitated cell survival both *in vitro* and *in vivo* mainly by promoting cell proliferation and inhibiting mitochondria-dependent intrinsic apoptosis, and thus contributed to poor prognosis of HCC patients. Mechanistically, the survival-promoting role of NPAS2 was mediated by transcriptional upregulation of the CDC25A phosphatase and subsequent dephosphorylation of CDK2/4/6 and Bcl-2, which induced cell proliferation and inhibited cell apoptosis in HCC, respectively. Moreover, BMAL1, another core clock transcription factor, was identified to heterodimerize with NPAS2 to bind to the E-box element in the promoter of *CDC25A* and be associated with the NPAS2-mediated tumor cell survival in HCC. Our findings demonstrate that NPAS2 has a critical role in HCC cell survival and tumor growth, which is mainly mediated by transcriptional upregulation of CDC25A. Thereby, NPAS2 may serve as a potential therapeutic target in HCC patients.

The circadian clock is a global regulatory system that generates rhythmic changes with 24-h periodicity in many important behaviors and physiological processes, including endocrine, metabolism and sleep/wake cycle.^1^ Increasing epidemiological studies have revealed a clear link between the disruption of circadian rhythms and tumor development, showing that shift workers have an increased risk of developing cancers of breast, colon, prostate, lung, ovarian and liver.^2, 3, 4^ In addition, the disruption of circadian machinery leads to changes in cellular functions such as metabolism and cell division, both highly relevant to cancer.^5, 6^ Moreover, the expression levels of circadian genes are associated with clinicopathological parameters in several cancers, and changes in the expression of those circadian genes can affect tumor growth, indicating an important role of the core circadian genes in carcinogenesis.^7^

It is well established that circadian rhythm is controlled by several core clock genes including *NPAS2, BMAL1, CLOCK, Per1/2/3* and *Cry1/2*.^6^ Several previous studies have addressed the relationship between neuronal PAS domain protein 2 (NPAS2) single-nucleotide polymorphisms (SNPs) and cancer risk.^8, 9, 10^ In addition, NPAS2 has also been identified to be a novel prognostic biomarker in breast and colorectal cancers.^11, 12^ Moreover, the silencing of NPAS2 expression promotes cell proliferation and invasion and increases the cell wound-healing ability in colorectal cancer, indicating a crucial tumor-suppressor role for NPAS2.^12^ Recently, we have demonstrated that functional polymorphisms in the *NPAS2* gene are associated with overall survival (OS) in transcatheter arterial chemoembolization-treated hepatocellular carcinoma (HCC) patients.^8^ However, to date, the potential functional roles of NPAS2 are greatly unclear in HCC. In this study, we systematically investigated the NPAS2 expression and its functional roles in HCC cell survival both *in vitro* and *in vivo*. More importantly, the underlying molecular mechanisms were deeply explored.

## Results

### Upregulation of NPAS2 is a frequent event in HCC tissues and associated with tumor progression and worse prognosis

**Figure 1:**
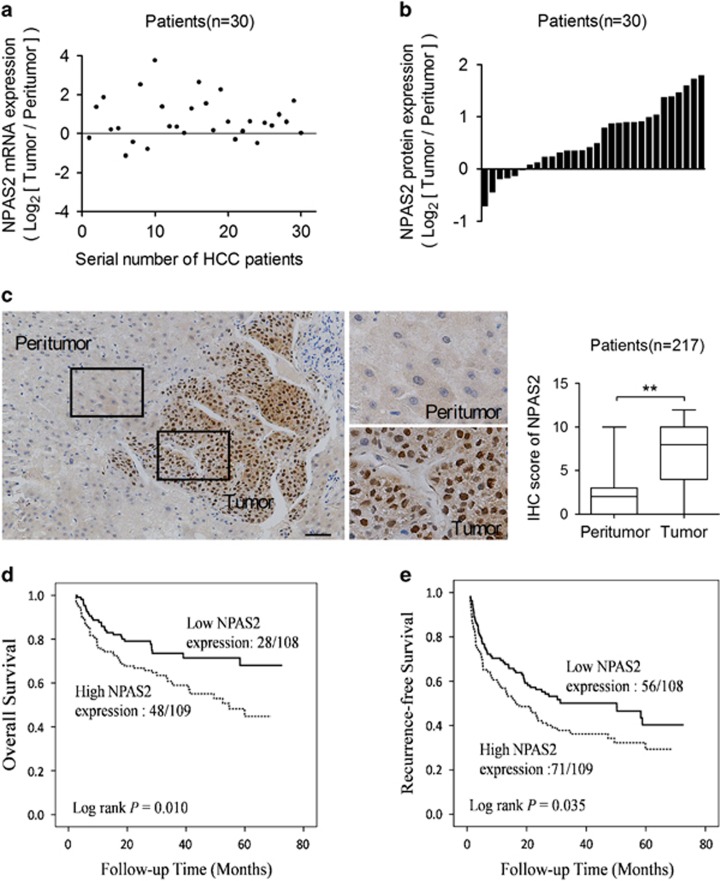
Upregulation of NPAS2 is a frequent event in HCC tissues and associated with tumor progression and worse prognosis. (**a** and **b**) qRT-PCR and western blot analyses for expression levels of NPAS2 in 30 paired tissues from HCC patients. T, tumor; P, peritumor. The relative expression ratio of tumor to peritumor was log_2_ transformed. The serial number of patient was rearranged for western blot according to expression level, whereas qRT-PCR data were displayed according to serial patient ID number. (**c**) Representative IHC staining images (left) and IHC scores (right) of NPAS2 in paired HCC tissues (*n*=217). *P<0.05; **P<0.01. Scale bar, 50 *μ*m. (**d** and **e**) Kaplan–Meier curve analysis of OS and RFS in HCC patients by the expression of NPAS2. Death/total and recurrence/total number of patients in each subgroup were presented

To determine the role of the positive limb of the core circadian genes in HCC, we first analyzed the mRNA expression level of the mammalian clock machinery genes (*NPAS2*, *BMAL1* and *CLOCK*) from four public data set from HCC patients and found that NPAS2 exhibited a significant upregulation in tumor tissues when compared with paired adjacent nontumor tissues (Supplementary Figure S1A). To validate this result, we assessed the expression level of NPAS2 in 30 paired HCC tissues. Quantitative reverse transcription-PCR (qRT)-PCR and western blot analyses showed that NPAS2 was significantly upregulated in HCC tumor tissues at both mRNA and protein levels when compared with paired nontumor tissues (Figures 1a and b and Supplementary Figure S1B). In addition, correlation analysis indicated a significant positive correlation between mRNA and protein expression levels for individual patients (Supplementary Figure S1C). Immunohistochemical (IHC) assay in a large cohort of 217 paired HCC tissues provided further support (Figure 1c). Moreover, our data indicated that high expression of NPAS2 was significantly associated with high AFP and larger tumor size (Supplementary Table 1), implying that NPAS2 may have a tumor-promoting role in HCC. Finally, Kaplan–Meier analysis revealed that HCC patients with high expression of NPAS2 had significantly poorer OS and recurrence-free survival (RFS) than those with low expression (log rank *P*=0.010 and 0.035, Figures 1d and e).

### NPAS2 promotes HCC cell survival *in vitro*

**Figure 2:**
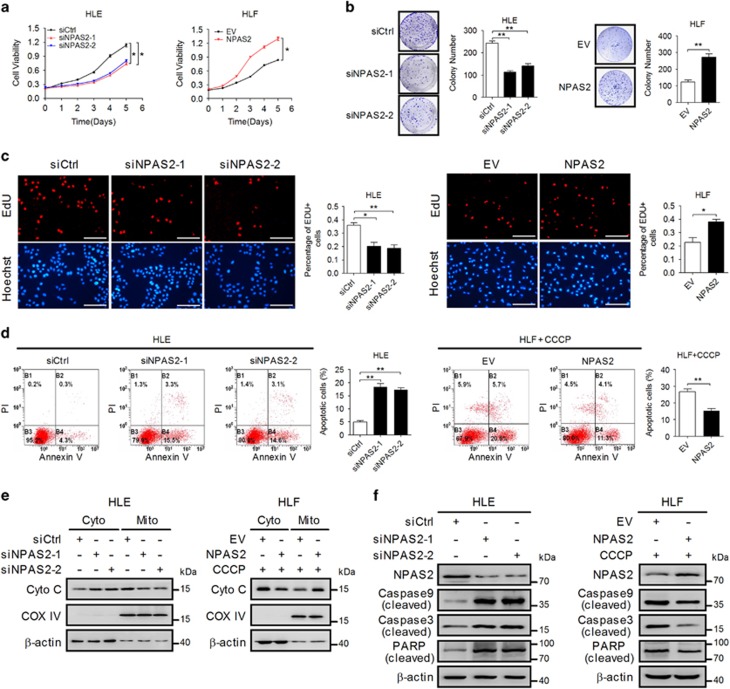
NPAS2 promotes HCC cell survival *in vitro*. (**a**) HCC cells were transiently transfected with siRNA or expression vector as indicated. Cells were reseeded for MTS cell viability assay 24 h after transfection. siNPAS2-1 and siNPAS2-2, siRNAs against NPAS2; siCtrl, control siRNA; NPAS2, expression vector encoding NPAS2; EV, empty vector. (**b**) Colony formation assay in HLE and HLF cells with treatment as indicated. (**c**) Cell proliferation ability was evaluated using EdU incorporation assay in HLE and HLF cells with treatment as indicated. Scale bar, 50 *μ*m. (**d**) Flow cytometry analysis of apoptosis by Annexin V (an indicator of apoptosis) and PI staining in both HLE and HLF cells 48 h after transfection with siRNA or expression vector as indicated. HLF cells were also treated with CCCP (150 *μ*M) for 4 h before apoptosis analysis. (**e**) Western blot analyses for protein levels of Cyto C in cytoplasm and mitochondria of HLE and HLF cells with treatment as indicated. *β*-Actin and COX IV were used as loading controls for cytoplasm and mitochondria, respectively. Cyto, cytoplasm; Mito, mitochondria. (**f**) Western blot analyses for protein levels of NPAS2, cleaved caspase 9, cleaved caspase 3 and cleaved PARP in HLE and HLF cells with treatment as indicated. Experiments were repeated twice with independent cell extracts and representative data were presented. The data shown are the mean±S.E.M. from three independent experiments. *P<0.05; **P<0.01

We further elucidated the functional role of NPAS2 in HCC cells. First, qRT-PCR and western blot analyses showed that the expressions of NPAS2 were significantly higher in six HCC cell lines than that in the non-transformed hepatic cell line (HL7702) (Supplementary Figure S2A). Moreover, HCC cells with relatively high (HLE) or low (HLF) NPAS2 expression were selected for the establishment of cell models with knockdown or forced expression of NPAS2, respectively (Supplementary Figures S2B and S2C). MTS and colony formation assays showed that HLE cells with NPAS2 knockdown had a much slower growth rate than control cells, whereas HLF cells with NPAS2 overexpression grew faster than control cells (Figures 2a and b). Considering that deregulated growth could be caused by uncontrolled proliferation and resistance to apoptosis, we evaluated the effect of NPAS2 on the ability of proliferation in HCC cells and found that EdU (5-ethynyl-2'-deoxyuridine) incorporation was significantly decreased in HLE cells with NPAS2 knockdown when compared with control cells, whereas the forced expression of NPAS2 in HLF cells exhibited an opposite effect (Figure 2c). Then, the potential functional role of NPAS2 in HCC cell apoptosis was explored. Our results showed that the percentage of total (both early and late) apoptotic cells were significantly higher in HLE cells with NPAS2 knockdown than that in control cells. In contrast, a lower percentage of total (both early and late) apoptotic cells, which is induced by CCCP (an uncoupler of oxidative phosphorylation in mitochondria), was observed in HLF cells with NPAS2 overexpression (Figure 2d). These results were further confirmed by a significant induction of cytochrome c (Cyto c, somatic) release and the cleavage of caspase 9 and caspase 3 in HLE cells with NPAS2 knockdown, while all of them were remarkably inhibited by NPAS2 overexpression in HLF cells upon CCCP treatment (Figures 2e and f). These observations collectively indicated that NPAS2 promoted cell survival *in vitro* mainly by accelerating cell proliferation and inhibiting cell apoptosis.

### NPAS2 promotes HCC growth *in vivo*

**Figure 3:**
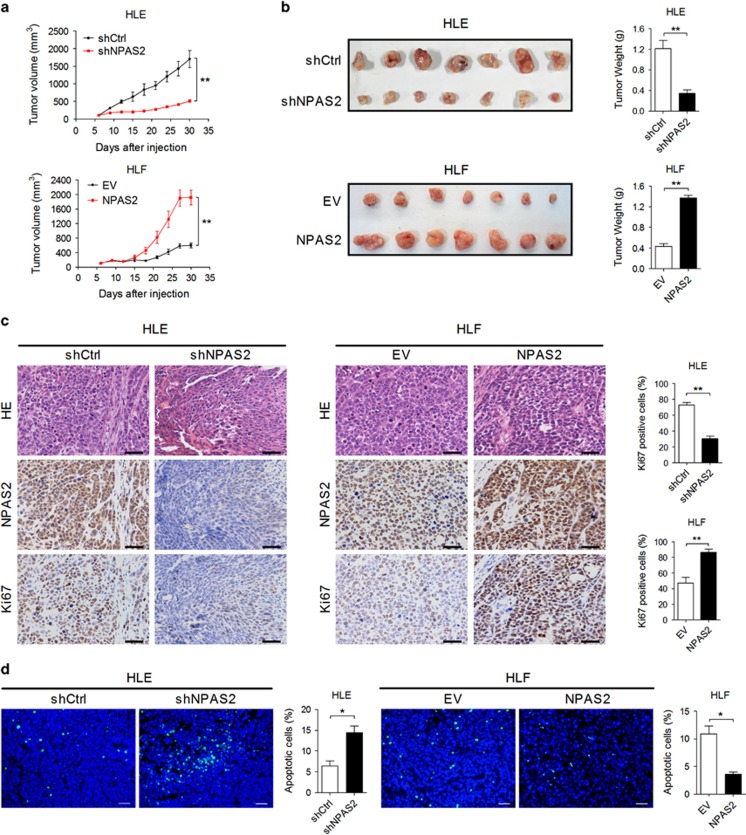
NPAS2 promotes HCC growth *in vivo*. (**a**) Tumor growth curves of subcutaneous xenograft tumor model developed from HCC cells, which were stably transfected with shRNA (*n*=7) or forced expression vector of NPAS2 (*n*=7) as indicated. Tumor size including tumor length (L) and width (W) was measured using vernier calipers every 3 days from day 6 after transplantation. The tumor volumes were calculated according to the formula (L × W^2^)/2 and presented as mean±S.E.M. Tumors from killed mice were dissected 30 days after transplantation. shNPAS2, shRNA expression vector against NPAS2; shCtrl, control shRNA. (**b**) Dissected tumors from killed mice were shown (left). The tumor weight was determined after tumor nodules were harvested (right). (**c**) Representative HE and IHC staining images of NPAS2 and Ki-67 in xenograft tumor treated as indicated (scale bar, 50 *μ*m) (left). Comparison of Ki-67-positive cells in tumor tissues of nude mice xenograft model with different treatment as indicated (right). (**d**) TUNEL staining in tumor tissues of nude mice xenograft model with different treatment as indicated. Blue: Hochest 33342; Green: TUNEL-positive nucleus (scale bar, 50 *μ*m). Data shown are the mean±S.E.M. from three independent experiments. *P<0.05; **P<0.01

We next examined the role of NPAS2 in HCC growth *in vivo* by constructing xenograft nude mice model using HCC cell lines with stable NPAS2 knockdown or overexpression (Supplementary Figures S2D and S2E). The stable knockdown of NPAS2 in HLE cells resulted in a significantly decreased tumor growth in xenograft model mice, whereas the growth capacity of xenograft tumors developed from HLF cells with stable overexpression of NPAS2 was much higher than control xenograft tumors (Figures 3a and b). Moreover, when compared with controls, those xenografts developed from HLE cells with NPAS2 stable knockdown exhibited a considerable decrease of positive Ki-67 staining and increase of positive TUNEL staining. In contrast, the forced expression of NPAS2 significantly increased Ki-67-positive staining and decreased TUNEL-positive staining in xenografts developed from HLF cells (Figures 3c and d). Taken together, these results show that NPAS2 promotes tumor growth *in vivo* by inducing cell proliferation and inhibiting cell apoptosis.

### NPAS2 transcriptionally upregulates CDC25A expression in HCC cells

**Figure 4:**
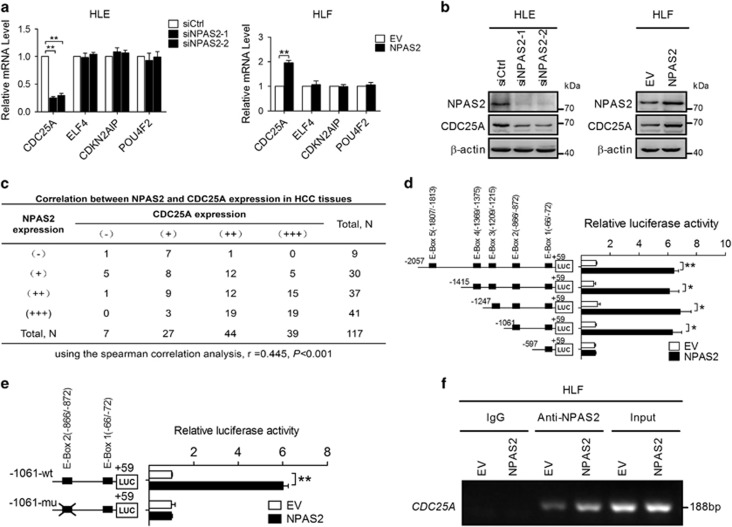
NPAS2 transcriptionally upregulates CDC25A expression in HCC cells. (**a**) qRT-PCR analyses for mRNA expression levels of CDC25A, ELF4, CDKN2AIP and POU4F2 in both HLE and HLF cells transiently transfected with siRNA or expression vector as indicated. (**b**) Western blot analyses for protein expression levels of CDC25A of HCC cells with treatment as indicated. Experiments were repeated twice with independent cell extracts and representative data were presented. (**c**) The correlation was evaluated between the protein expression levels of NPAS2 and CDC25A in 117 HCC tissues based on IHC staining. (**d**) HLF cells were transfected with constructs with serially truncated CDC25A promoter constructs, and the cells were treated with or without ectopic expression of NPAS2 as indicated. Twenty-four hours after treatment, the luciferase activity was determined. (**e**) HLF cells were transfected with −1061/+59 CDC25A-wt or selective mutation (−1061/+59) CDC25A-mu promoter constructs. (**f**) Amplification of the CDC25A promoter sequence from ChIP DNA was performed (HLF). Input and IgG served as positive and negative controls, respectively. The electrophoresis results of PCR products confirmed that NPAS2 binds to the CDC25A promoter. Data shown are the mean±S.E.M. from three independent experiments. *P<0.05; **P<0.01

To explore the mechanism by which NPAS2 affects cell proliferation and apoptosis, we attempted to identify the potential target genes of NPAS2 in HCC cells. A previous study using the genome-wide ChIP-on-chip analysis has identified 16 genes as direct transcriptional targets of NPAS2 in breast cancer cells, among which 4 genes including *CDC25A*, *ELF4*, *CDKN2AIP* and *POU4F2* were involved in cell proliferation and apoptosis regulation.^13^ Therefore, functional roles of NPAS2 in the transcriptional regulation of these genes were determined in HCC cells. We found that both mRNA and protein levels of CDC25A were significantly decreased in HLE cells with NPAS2 knockdown and were significantly increased in HLF cells with NPAS2 overexpression (Figures 4a and b). In contrast, the expression of ELF4, CDKN2AIP and POU4F2 was not affected by NPAS2. To provide further support, we detected the expression of both NPAS2 and CDC25A in HCC tissues (Supplementary Figure S3A). Spearman rank correlation analysis indicated a significant positive correlation between IHC scores of NPAS2 and CDC25A (*r*=0.445, *P*<0.001) (Figure 4c). Moreover, bioinformatic analysis based on four public mRNA expression data set also showed a significant positive correlation between NPAS2 and CDC25A expression (Supplementary Figure S3C).

Considering that NPAS2 acts primarily as a transcription factor, we hypothesized that CDC25A may be a transcriptional target of NPAS2. To test it, we analyzed the DNA sequence of CDC25A promoter and identified five E-boxes, which are putative DNA-binding sites for NPAS2 (Figure 4d). Serial deletion and site-directed mutagenesis analyses showed that the second NPAS2 binding site was critical for NPAS2-induced CDC25A transactivation (Figures 4d and e). ChIP assay further confirmed that NPAS2 binds directly to the CDC25A promoter in HLF and HLE cells (Figure 4f and Supplementary Figure S2F). Collectively, these results show that NPAS2 directly binds to the E-box site in the CDC25A promoter region from nt-872 to nt-866 to transactivate CDC25A mRNA expression.

### NPAS2 promotes HCC cell survival through upregulating CDC25A

**Figure 5:**
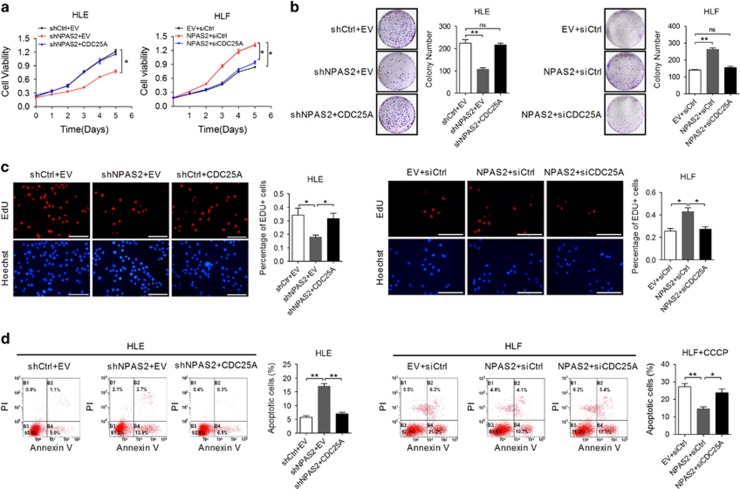
NPAS2 promotes HCC cell survival through upregulating CDC25A. **(a**) Cell viability was evaluated by MTS assay in HCC cells, which were stably transfected with shRNA or forced expression vector. shNPAS2, shRNA expression vector against NPAS2; shCtrl, control shRNA. CDC25A, expression vector encoding CDC25A. (**b**) Colony formation assay in HLE and HLF cells treated as indicated. (**c**) EdU incorporation assay in HCC cells treated as indicated. Scale bar, 50 *μ*m. (**d**) Cell apoptosis analysis in HCC cells with a panel of treatment as indicated. Data shown are the mean±S.E.M. from three independent experiments. *P<0.05; **P<0.01

It is well established that CDC25A is involved in both apoptosis and cell cycle regulation.^14^ Therefore, it is reasonable to hypothesize that NPAS2 may promote HCC cell survival by modulating transcription of CDC25A. To test it, CDC25A was overexpressed in HLE cells with NPAS2 stable knockdown. As shown in Figures 5a and b, overexpression of CDC25A significantly reverted the cell growth rate and colony formation ability, which was decreased by knockdown of NPAS2 in HLE cells. In contrast, the silencing of CDC25A significantly attenuated the growth rate and colony formation efficiency, which was increased by NPAS2 stable overexpression in HLF cells. Furthermore, we found that the overexpression of CDC25A lead to an increased cell proliferation and a decreased cell apoptosis in HLE cells with NPAS2 stable knockdown, whereas silencing of CDC25A exhibited an opposite effect in HLF cells with NPAS2 stable overexpression (Figures 5c and d). Taken together, these results indicate that CDC25A is a crucial molecule involved in the effect of NPAS2 on cell growth in HCC cells.

### NPAS2 promotes G1 to S phase transition of cell cycle by CDC25A-mediated activation of CDK2/4/6

**Figure 6:**
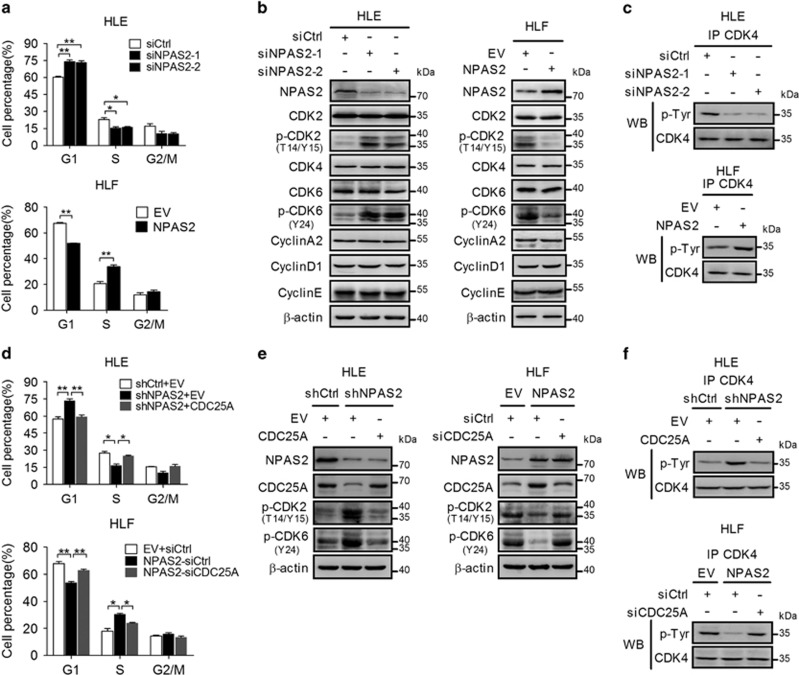
NPAS2 promotes G1 to S phase transition of cell cycle by CDC25A-mediated activation of CDK2/4/6. (**a**) Cell cycle analysis by flow cytometry in HLE and HLF cells after transiently transfected with siRNA or expression vector as indicated. The percentage of cell population at different cell cycle phases are shown in the histograms and statistically analyzed using a two-tailed *t*-test. (**b**) Western blot analyses for NPAS2, CDK2, phosphorylated CDK2 at T14/Y15 (p-CDK2), CDK4, CDK6, phosphorylated CDK6 at Y24 (p-CDK6), cyclin A2, cyclin D1 and cyclin E in HLE and HLF cells treated as indicated. (**c**) HLE and HLF cells transfected with siRNA or expression vector as indicated. Tyrosine phosphorylation (P-Tyr) was detected by western blot after IP with anti-CDK4 antibodies. (**d**) Cell cycle analysis by flow cytometry was performed in HLE and HLF cells with treatment as indicated. (**e**) Western blot analyses for NPAS2, CDC25A, p-CDK2 (T14/Y15), p-CDK6 (Y24) in HLE and HLF cells with treatment as indicated. (**f**) Tyrosine phosphorylation (P-Tyr) was detected by western blot after IP with anti-CDK4 antibodies in HLE and HLF cells with treatment as indicated. Experiments were repeated twice with independent cell extracts and representative data were presented. Data shown are the mean±S.E.M. from three independent experiments. *P<0.05; **P<0.01

To gain mechanistic insight on how NPAS2 accelerates cell proliferation through upregulating CDC25A, cell cycle analysis by flow cytometry was performed. Our data showed that HLE cells with NPAS2 knockdown exhibited a significant accumulation in G1 phase and a remarkable decrease in S phases, whereas NPAS2 overexpression significantly decreased the percentage of HLF cells in G1 phase and increased that in S phase (Figure 6a and Supplementary Figure S4A), suggesting that NPAS2 promotes S phase entry in HCC cells. To further investigate the molecular mechanism, we first evaluated the expression of key cell cycle components involved in the G1 to S transition. Our results showed that the inhibitory phosphorylation of CDK2(T14/Y15) and CDK6(Y24) was significantly increased by NPAS2 knockdown and decreased by NPAS2 overexpression (Figure 6b). Also, CDK4 immunoprecipitations (IPs) coupled to anti-phosphotyrosine immunoblotting also demonstrated that CDK4 was significantly tyrosine phosphorylated by NPAS2 knockdown and strongly dephosphorylated upon NPAS2 overexpression (Figure 6c).^14^ Next, we investigated whether CDC25A-mediated dephosphorylation of CDK2(T14/Y15), CDK4(Y) and CDK6(Y24) is involved in NPAS2-induced G1 to S phase transition. The cell cycle assay showed that overexpression of CDC25A rescued the accumulation of cells in G1 phase induced by NPAS2 stable knockdown, whereas CDC25A silencing by siRNA increased the percentage of HCC cells in G1 phase induced by NPAS2 stable overexpression (Figure 6d and Supplementary Figure S4B). In addition, we also observed a remarkably decreased phosphorylation of CDK2 (T14/Y15), CDK4(Y) and CDK6 (Y24) upon CDC25A overexpression and a significant increase upon CDC25A knockdown (Figures 6e and f). Collectively, these datademonstrate that NPAS2 promotes G1 to S phase transition of cell cycle by CDC25A-mediated activation of CDK2/4/6.

### Dephosphorylation of Bcl-2 at Thr69 by CDC25A is required in inhibition of apoptosis by NPAS2

**Figure 7:**
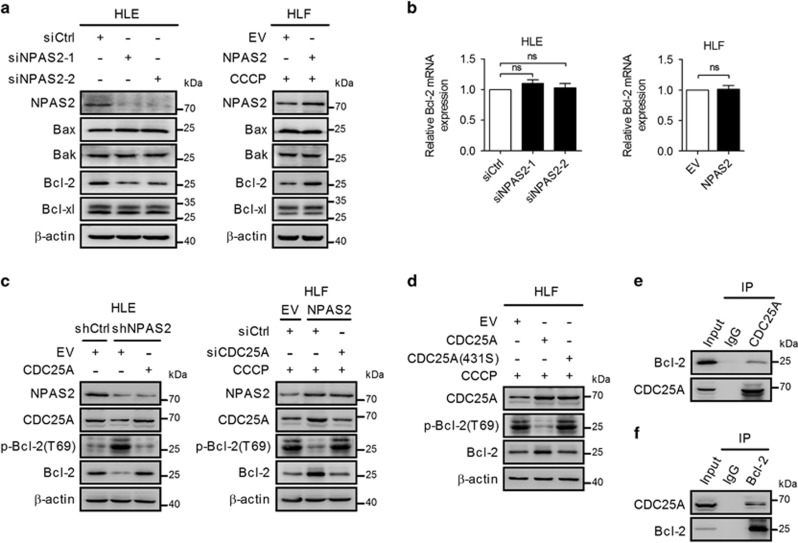
Dephosphorylation of Bcl-2 at Thr69 by CDC25A is required in inhibition of apoptosis by NPAS2. (**a**) Western blot analyses for NPAS2, Bax, Bak, Bcl-2 and Bcl-xl in HLE and HLF cells with treatment as indicated. (**b**) qRT-PCR analyses for mRNA expression levels of Bcl-2 in both HLE and HLF cells with treatment as indicated. (**c**) Western blot analyses for NPAS2, CDC25A, phosphorylated Bcl-2 at T69 (p-Bcl-2) and Bcl-2 in HLE and HLF cells treated as indicated. (**d**) Western blot analyses for CDC25A, p-Bcl-2 (T69) and Bcl-2 in HLF cells transiently transfected with a control vector, or CDC25A, or phosphatase-inactive CDC25A (C431S). (**e and f**) Co-IP assay using control IgG and anti-CDC25A or anti-Bcl-2 antibody was carried out using extracts prepared from HLF cells. The presence of CDC25A or Bcl-2 in these IPs was evaluated by immunoblotting (WB). Experiments were repeated twice with independent cell extracts and representative data were presented. Data shown are the mean±S.E.M. from three independent experiments. *P<0.05; **P<0.01

To investigate the molecular mechanism by which NPAS2 inhibits HCC cell apoptosis, we examined the expression of pivotal proteins involved in the cell apoptosis. Western blot analyses showed a significant reduction of a key anti-apoptotic protein Bcl-2 in HLE cells with NPAS2 knockdown and a significant increase of Bcl-2 expression in HLF cells with NPAS2 overexpression. However, the expression of pro-apoptosis protein Bax, Bak and anti-apoptotic protein Bcl-xl was not affected (Figure 7a). Quantitative RT-PCRs were performed to distinguish between transcriptional and posttranscriptional regulation of Bcl-2 by NPAS2. Our data showed that the mRNA level of Bcl-2 was not changed by either NPAS2 knockdown or overexpression, excluding the possibility of Bcl-2 as a direct transcriptional target of NPAS2 (Figure 7b).

It is well established that three phosphorylation sites (Thr69, Ser87 and Ser70) of Bcl-2 are involved in its degradation.^15, 16^ Considering that CDC25A is a dual specificity phosphatases that dephosphorylate both threonine and tyrosine residues,^17^ we examined the potential phosphorylation target of Bcl-2 at Thr69 site. Western blot analyses showed that Bcl-2 T69 phosphorylation was markedly decreased upon overexpression of NPAS2 or CDC25A in HCC cells, whereas the phosphorylation of Bcl-2 T69 was significantly enhanced in HLE cells with NPAS2 or CDC25A knockdown (Figure 7c). To further confirm if the Bcl-2 T69 phosphorylation is dependent on the phosphatase activity of CDC25A, we treated HLF cells with recombinant wild-type CDC25A or catalytically inactive (C431S) forms of CDC25A.^18^ Overexpression of wild-type CDC25A suppressed the phosphorylation of Bcl-2 T69 and increased the expression level of Bcl-2, whereas overexpression of phosphatase-inactive CDC25A (C431S) had no effect on the levels of Bcl-2 and Bcl-2 T69 phosphorylation (Figure 7d). Moreover, co-immunoprecipitation (co-IP) assays showed that CDC25A and Bcl-2 formed a protein complex in HLE and HLF cells, which was IP with anti-CDC25A or anti-Bcl-2 antibodies, followed by detection with anti-Bcl-2 or anti-CDC25A antibodies (Figures 7e,Supplementary Figure S2G). Taken together, these data indicate that dephosphorylation of Bcl-2 at Thr69 by CDC25A is required in inhibition of apoptosis by NPAS2.

### NPAS2 in association with BMAL1 promotes cell survival of HCC cells

**Figure 8:**
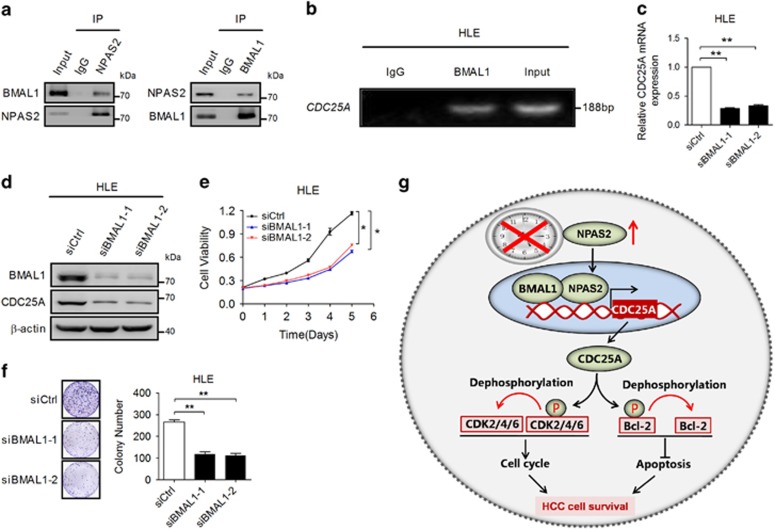
NPAS2 in association with BMAL1 promotes cell survival of HCC cells. (**a**) Co-IP assay was performed with anti-NPAS2 or anti-BMAL1 antibody was carried out using extracts prepared from HLE cells. The presence of BMAL1 or NPAS2 in these IPs was evaluated by immunoblotting (WB). (**b**) Amplification of the CDC25A promoter sequence from ChIP DNA was performed (HLE). Input and IgG served as positive and negative controls, respectively. (**c** and **d**) qRT-PCR and western blot analyses for CDC25A expression were performed in HLE cells treated with siRNA against BMAL1. (**e**) HLE cells were transiently transfected with siRNA as indicated. Cells were reseeded for MTS cell viability assay 24 h after transfection. (**f**) Colony formation assay in HLE cells treated with siRNA against BMAL1. (**g**) Schematic depicting the regulation of cell survival by NPAS2 in HCC. Experiments were repeated twice with independent cell extracts and representative data were presented. Data shown are the mean±S.E.M. from three independent experiments. *P<0.05; **P<0.01

NPAS2 forms heterodimer with another core circadian rhythm transcription factor BMAL1 to control the expression of targeted genes in mouse forebrain and vascular cells.^19, 20^ Therefore, we investigated whether BMAL1 is involved in the functional effect of NPAS2 on HCC cell survival. Co-IP assays showed that BMAL1 heterodimerize with NPAS2 in HCC cells (Figure 8a and Supplementary Figure S4C). To further determine whether NPAS2 and BMAL1 co-binds to the CDC25A promoter, we performed ChIP-PCR assay in HCC cells and observed that BMAL1 directly binds to the promoter of CDC25A (Figure 8b and Supplementary Figure S4D), suggesting an essential role of BMAL1 in NPAS2-mediated transactivation of CDC25A. Moreover, BMAL1 knockdown robustly reduced the expression of CDC25A both at mRNA and protein levels as expected (Figures 8c and d). The knockdown of BMAL1 expression in HLE was confirmed by qRT-PCR and western blot analyses (Supplementary Figures S4E and S4F). We further determined whether BMAL1 was involved in NPAS2-modulated HCC cell growth. MTS and colony formation assays indicated that BMAL1 knockdown phenocopied NPAS2 knockdown, which showed a significantly slower rate of cell growth (Figures 8e and f). Collectively, these data suggest that NPAS2 controls the expression of CDC25A by forming heterodimer with BMAL1 to promote HCC cell survival.

## Discussion

Despite several lines of evidence implicating circadian rhythm disruption in cancer, little is known about the roles of the core circadian genes in tumor initiation and progression and its underlying mechanism, especially in HCC. In this study, we demonstrated that NPAS2 was frequently upregulated in HCC, which significantly facilitated cell survival both *in vitro* and *in vivo* mainly by promoting cell proliferation and inhibiting mitochondria-dependent intrinsic apoptosis, and thus contributed to poor prognosis of HCC patients. Mechanistically, we demonstrated that the survival-promoting role of NPAS2 was mediated via transcriptional upregulation of the CDC25A phosphatase and subsequent dephosphorylation of CDK2/4/6 and Bcl-2. Moreover, another core circadian gene BMAL1 was also found to be associated with the NPAS2-mediated tumor cell survival in HCC (Figure 8g).

Several previous studies have reported that NPAS2 acts as a tumor suppressor in colorectal and breast cancers. For example, Xue *et al.*^12^ have showed that NPAS2 mRNA expression is reduced in colorectal cancer and silencing of NPAS2 expression promotes cell proliferation, cell invasion and increases the wound-healing ability of colorectal cancer cells. Yi *et al.* have reported that high level of NPAS2 mRNA expression is associated with increased disease free and OSs in breast cancer.^11^ In contrast, our data have demonstrated that NPAS2 is frequently upregulated in HCC cell lines and tissues and high expression of NPAS2 is associated with the aggressive clinical characteristics and poor prognosis in HCC patients. In addition, functional analysis supports a tumor-promoting function for NPAS2 in HCC. These contradictions may be explained by the fact that the expression level and function of NPAS2 is tumor type specific, which was further supported by our analysis for the prognostic value of NPAS2 in multiple types of cancer from the PrognoScan (a database for meta-analysis of the prognostic value of genes) (Supplementary Table 5), indicating that NPAS2 expression is a favorable prognostic factor in breast and ovarian cancer but is a poor prognosis factor in lung, soft tissue, brain and skin cancers.

Previous studies have showed that downregulated NPAS2 expression led to the cell cycle arrest at S and G2/M phase in colorectal cancer cells.^12^ NPAS2 silencing results in aberrant cell cycle response to DNA damage in breast cancer cells.^21^ In this study, we demonstrated that NPAS2 significantly facilitated cell survival both *in vitro* and *in vivo* by promoting cell cycle G1-S transition. The opposing effects of NPAS2 in cell cycle could be explained by the fact that the function of NPAS2 is tumor type specific. In addition, it has recently been well documented that circadian genes have important roles in regulating cell apoptosis in various cancer cells.^22, 23, 24, 25^ Our present study showed that NPAS2 overexpression inhibits apoptosis of HCC cells. Similarly, a previous study has indicated that BMAL1 depletion increases apoptotic cell population in malignant pleural mesothelioma.^22^ However, previous studies by Xue *et al* and Hoffman *et al* have suggested that NPAS2 silencing does not influence the apoptotic rate in colorectal and breast cancer cells.^12, 21^ Possible explanations for these differences include different tumor origins and various apoptotic mechanisms.

CDC25A, a member of the CDC25 family of phosphatases, is highly expressed in various malignancies including HCC and proved to be associated with poor survival,^26, 27^ which is highly consistent with our result that tumor tissues from HCC patients exhibited the upregulated CDC25A expression (Supplementary Figure S3B). However, the detailed mechanism underlying CDC25A overexpression in cancer cells is still unknown. Here, we demonstrated that NPAS2 transcriptionally activated CDC25A in HCC cells through direct binding to the CDC25A promoter region at sites nt-872 to nt-866. CDC25A is known as an oncogene to assist both G1/S and G2/M progression in various types of cancers including HCC.^26, 27, 28^ Our present study showed that NPAS2 facilitated G1-S transition via transcriptional upregulation of the CDC25A and subsequent dephosphorylates CDK4(Tyr), CDK6(Tyr24) and CDK2(Thr14/Tyr15) in HCC, strongly suggesting that CDC25A is a vital bridge between circadian rhythm and cell cycle regulation. However, the functional role of CDC25A as an important phosphatase in the regulation of apoptosis is still unclear. Bcl-2 is a key regulator of cell apoptosis and a body of evidence has indicated that the abundance and function of Bcl-2 are influenced by posttranslational modifications. Previous studies have demonstrated that the dephosphorylation of Bcl-2 at Thr69 increases Bcl-2 stability and thus inhibits cell apoptosis.^15, 29^ In this study, we demonstrated that NPAS2 inhibited apoptosis of HCC cells through transactivation of CDC25A and subsequent dephosphorylation of Bcl-2(Thr69).

In the core oscillator, NPAS2/BMAL1 heterodimer directly binds to the E-box and thus drive transcription of a number of clock genes.^30, 31^ In this study, we demonstrated that BMAL1 knockdown robustly reduced the expression of CDC25A and phenocopied the functional role of NPAS2 in HCC cell survival, indicating the importance of NPAS2/BMAL1 heterodimer in HCC progression. Similar result has been reported in non-transformed cells of HEK293.^32^ Thus, the decomposition of the NPAS2/BMAL1 heterodimer may provide a potential strategy for HCC targeting therapy.

In summary, we show a key pro-survival role of NPAS2 in HCC and provide a comprehensive view of underlying molecular mechanism. Our study suggest a possibility that NPAS2 may be used as a therapeutic target in HCC.

## Materials and Methods

### Cell culture, tissue collection and public data set collection

Human HCC cell lines HLE and HLF were obtained from Japanese Collection of Research Bioresources (Osaka, Japan) and authenticated using short tandem repeat DNA testing by the FMMU Center for DNA Typing in 2016. Both cells were routinely cultured. In addition, 217 human HCC tissue samples were obtained as described in Supplementary Methods. Moreover, four public data sets of mRNA expression from human HCC tissues were analyzed, including RNA-seq data from the Cancer Genome Atlas (TCGA) and microarray data of GSE14520, GSE22058 and GSE25097 from Gene Expression Omnibus (GEO) database. The detailed information was listed in Supplementary Table 2.

### qRT-PCR, western blot and IHC

RNA extraction, complementary DNA synthesis and qRT-PCR reactions were performed as described in Supplementary Methods. Primer sequences used in this study were provided in Supplementary Table 3. HCC tissues and cell lines were processed for western blot and IHC as previously described.^33^ Quantification of western blot bands and IHC staining scoring were performed as described in Supplementary Methods. The primary antibodies used in this study and their working concentration were listed in Supplementary Table 4.

### Nude mice xenograft model

Nude mice xenograft model was used to assess *in vivo* tumor growth as described in Supplementary Methods.

### Detection of cell viability, cell proliferation, cell apoptosis and cell cycle

Cell viability was determined by the MTS (Promega Corp., Hercules, CA, USA) and colony formation assays as described in Supplementary Methods. The EdU incorporation assay kit (Ribobio, Guangdong, China) was used for cell proliferation analysis according to the manufacturer's instructions. The apoptosis rate and cell cycle were respectively determined by flow cytometry using Annexin V-FITC Apoptosis Detection Kit (BestBio, Shanghai, China) and PI staining (BestBio) following the manufacturers' instructions. TUNEL assay was performed for apoptosis analysis in xenograft tissues as described in Supplementary Methods.

### Construction of reporter plasmids and site-directed mutagenesis

Construction of reporter plasmids and site-directed mutagenesis were performed as described in Supplementary Methods.

### Luciferase assay

The luciferase assays were performed using the above-mentioned constructs, and the detailed description of the luciferase assays appears in Supplementary Methods.

### Chromatin immunoprecipitation (ChIP)-PCR assays, IP and co-IP assays

The detailed procedures were described in Supplementary Methods.

### Statistical analysis

SPSS 17.0 software (SPSS, Chicago, IL, USA) was used for all statistical analyses and detailed method was provided in Supplementary Methods.

## References

[bib1] Milev NB, Reddy AB. Circadian redox oscillations and metabolism. Trends Endocrinol Metab 2015; 26: 430–437. 26113283 10.1016/j.tem.2015.05.012 PMC5122445

[bib2] Straif K, Baan R, Grosse Y, Secretan B, El GF, Bouvard V et al. Carcinogenicity of shift-work, painting, and fire-fighting. Lancet Oncol 2007; 8: 1065–1066. 19271347 10.1016/S1470-2045(07)70373-X

[bib3] Davis S, Mirick DK, Stevens RG. Night shift work, light at night, and risk of breast cancer. J Natl Cancer Inst 2001; 93: 1557–1562. 11604479 10.1093/jnci/93.20.1557

[bib4] Baan R, Grosse Y, Straif K, Secretan B, El GF, Bouvard V et al. A review of human carcinogens--part F: chemical agents and related occupations. Lancet Oncol 2009; 10: 1143–1144. 19998521 10.1016/s1470-2045(09)70358-4

[bib5] Bass J, Takahashi JS. Circadian integration of metabolism and energetics. Science 2010; 330: 1349–1354. 21127246 10.1126/science.1195027 PMC3756146

[bib6] Sahar S, Sassone-Corsi P. Metabolism and cancer: the circadian clock connection. Nat Rev Cancer 2009; 9: 886–896. 19935677 10.1038/nrc2747

[bib7] Kelleher FC, Rao A, Maguire A. Circadian molecular clocks and cancer. Cancer Lett 2014; 342: 9–18. 24099911 10.1016/j.canlet.2013.09.040

[bib8] Yuan P, Wang S, Zhou F, Wan S, Yang Y, Huang X et al. Functional polymorphisms in the NPAS2 gene are associated with overall survival in transcatheter arterial chemoembolization-treated hepatocellular carcinoma patients. Cancer Sci 2014; 105: 825–832. 24754267 10.1111/cas.12428 PMC4317913

[bib9] Rana S, Shahid A, Ullah H, Mahmood S. Lack of association of the NPAS2 gene Ala394Thr polymorphism (rs2305160:G>A) with risk of chronic lymphocytic leukemia. Asian Pac J Cancer Prev 2014; 15: 7169–7174. 25227809 10.7314/apjcp.2014.15.17.7169

[bib10] Madden MH, Anic GM, Thompson RC, Nabors LB, Olson JJ, Browning JE et al. Circadian pathway genes in relation to glioma risk and outcome. Cancer Causes Control 2014; 25: 25–32. 24135790 10.1007/s10552-013-0305-y PMC3947318

[bib11] Yi C, Mu L, de la Longrais IA, Sochirca O, Arisio R, Yu H et al. The circadian gene NPAS2 is a novel prognostic biomarker for breast cancer. Breast Cancer Res Treat 2010; 120: 663–669. 19649706 10.1007/s10549-009-0484-0 PMC3108061

[bib12] Xue X, Liu F, Han Y, Li P, Yuan B, Wang X et al. Silencing NPAS2 promotes cell growth and invasion in DLD-1 cells and correlated with poor prognosis of colorectal cancer. Biochem Biophys Res Commun 2014; 450: 1058–1062. 24978311 10.1016/j.bbrc.2014.06.104

[bib13] Yi CH, Zheng T, Leaderer D, Hoffman A, Zhu Y. Cancer-related transcriptional targets of the circadian gene NPAS2 identified by genome-wide ChIP-on-chip analysis. Cancer Lett 2009; 284: 149–156. 19457610 10.1016/j.canlet.2009.04.017 PMC3182267

[bib14] Shen T, Huang S. The role of Cdc25A in the regulation of cell proliferation and apoptosis. Anticancer Agents Med Chem 2012; 12: 631–639. 22263797 10.2174/187152012800617678 PMC3544488

[bib15] Lin SS, Bassik MC, Suh H, Nishino M, Arroyo JD, Hahn WC et al. PP2A regulates BCL-2 phosphorylation and proteasome-mediated degradation at the endoplasmic reticulum. J Biol Chem 2006; 281: 23003–23012. 16717086 10.1074/jbc.M602648200

[bib16] Kutuk O, Letai A. Regulation of Bcl-2 family proteins by posttranslational modifications. Curr Mol Med 2008; 8: 102–118. 18336291 10.2174/156652408783769599

[bib17] Brenner AK, Reikvam H, Lavecchia A, Bruserud O. Therapeutic targeting the cell division cycle 25 (CDC25) phosphatases in human acute myeloid leukemia--the possibility to target several kinases through inhibition of the various CDC25 isoforms. Molecules 2014; 19: 18414–18447. 25397735 10.3390/molecules191118414 PMC6270710

[bib18] Bertero T, Gastaldi C, Bourget-Ponzio I, Mari B, Meneguzzi G, Barbry P et al. CDC25A targeting by miR-483-3p decreases CCND-CDK4/6 assembly and contributes to cell cycle arrest. Cell Death Differ 2013; 20: 800–811. 23429262 10.1038/cdd.2013.5 PMC3647239

[bib19] Reick M, Garcia JA, Dudley C, McKnight SL. NPAS2: an analog of clock operative in the mammalian forebrain. Science 2001; 293: 506–509. 11441147 10.1126/science.1060699

[bib20] McNamara P, Seo SB, Rudic RD, Sehgal A, Chakravarti D, FitzGerald GA. Regulation of CLOCK and MOP4 by nuclear hormone receptors in the vasculature: a humoral mechanism to reset a peripheral clock. Cell 2001; 105: 877–889. 11439184 10.1016/s0092-8674(01)00401-9

[bib21] Hoffman AE, Zheng T, Ba Y, Zhu Y. The circadian gene NPAS2, a putative tumor suppressor, is involved in DNA damage response. Mol Cancer Res 2008; 6: 1461–1468. 18819933 10.1158/1541-7786.MCR-07-2094 PMC2572675

[bib22] Elshazley M, Sato M, Hase T, Yamashita R, Yoshida K, Toyokuni S et al. The circadian clock gene BMAL1 is a novel therapeutic target for malignant pleural mesothelioma. Int J Cancer 2012; 131: 2820–2831. 22510946 10.1002/ijc.27598 PMC3479344

[bib23] Jiang W, Zhao S, Jiang X, Zhang E, Hu G, Hu B et al. The circadian clock gene Bmal1 acts as a potential anti-oncogene in pancreatic cancer by activating the p53 tumor suppressor pathway. Cancer Lett 2016; 371: 314–325. 26683776 10.1016/j.canlet.2015.12.002

[bib24] Li HX, Fu XJ, Yang K, Chen D, Tang H, Zhao Q. The clock gene PER1 suppresses expression of tumor-related genes in human oral squamous cell carcinoma. Oncotarget 2016; 7: 20574–20583. 26943040 10.18632/oncotarget.7827 PMC4991476

[bib25] Cao Q, Gery S, Dashti A, Yin D, Zhou Y, Gu J et al. A role for the clock gene per1 in prostate cancer. Cancer Res 2009; 69: 7619–7625. 19752089 10.1158/0008-5472.CAN-08-4199 PMC2756309

[bib26] Boutros R, Lobjois V, Ducommun B. CDC25 phosphatases in cancer cells: key players? Good targets? Nat Rev Cancer 2007; 7: 495–507. 17568790 10.1038/nrc2169

[bib27] Xu X, Yamamoto H, Sakon M, Yasui M, Ngan CY, Fukunaga H et al. Overexpression of CDC25A phosphatase is associated with hypergrowth activity and poor prognosis of human hepatocellular carcinomas. Clin Cancer Res 2003; 9: 1764–1772. 12738732

[bib28] Bartek J, Lukas J. Mammalian G1- and S-phase checkpoints in response to DNA damage. Curr Opin Cell Biol 2001; 13: 738–747. 11698191 10.1016/s0955-0674(00)00280-5

[bib29] Yamamoto K, Ichijo H, Korsmeyer SJ. BCL-2 is phosphorylated and inactivated by an ASK1/Jun N-terminal protein kinase pathway normally activated at G(2)/M. Mol Cell Biol 1999; 19: 8469–8478. 10567572 10.1128/mcb.19.12.8469 PMC84954

[bib30] Fukuhara C, Liu C, Ivanova TN, Chan GC, Storm DR, Iuvone PM et al. Gating of the cAMP signaling cascade and melatonin synthesis by the circadian clock in mammalian retina. J Neurosci 2004; 24: 1803–1811. 14985420 10.1523/JNEUROSCI.4988-03.2004 PMC6730387

[bib31] Jin X, Shearman LP, Weaver DR, Zylka MJ, de Vries GJ, Reppert SM. A molecular mechanism regulating rhythmic output from the suprachiasmatic circadian clock. Cell 1999; 96: 57–68. 9989497 10.1016/s0092-8674(00)80959-9

[bib32] Garcia JA. Impaired cued and contextual memory in NPAS2-deficient mice. Science 2000; 288: 2226–2230. 10864874 10.1126/science.288.5474.2226

[bib33] Li J, Huang Q, Long X, Zhang J, Huang X, Aa J et al. CD147 reprograms fatty acid metabolism in hepatocellular carcinoma cells through Akt/mTOR/SREBP1c and P38/PPARalpha pathways. J Hepatol 2015; 63: 1378–1389. 26282231 10.1016/j.jhep.2015.07.039

