# Supplementary material for: NPAS2 promotes cell survival of hepatocellular carcinoma by transactivating CDC25A
Source: Cell Death Dis. 2017 Mar 23;8(3):e2704–. doi: 10.1038/cddis.2017.131 (PMC5386534; doi:10.1038/cddis.2017.131)
Supplement: Supplementary Information [file cddis2017131x1.doc]

**Supplementary information**

**NPAS2 Promotes Cell Survival of Hepatocellular Carcinoma by Transactivating CDC25A**

**Supplementary Figures**

**
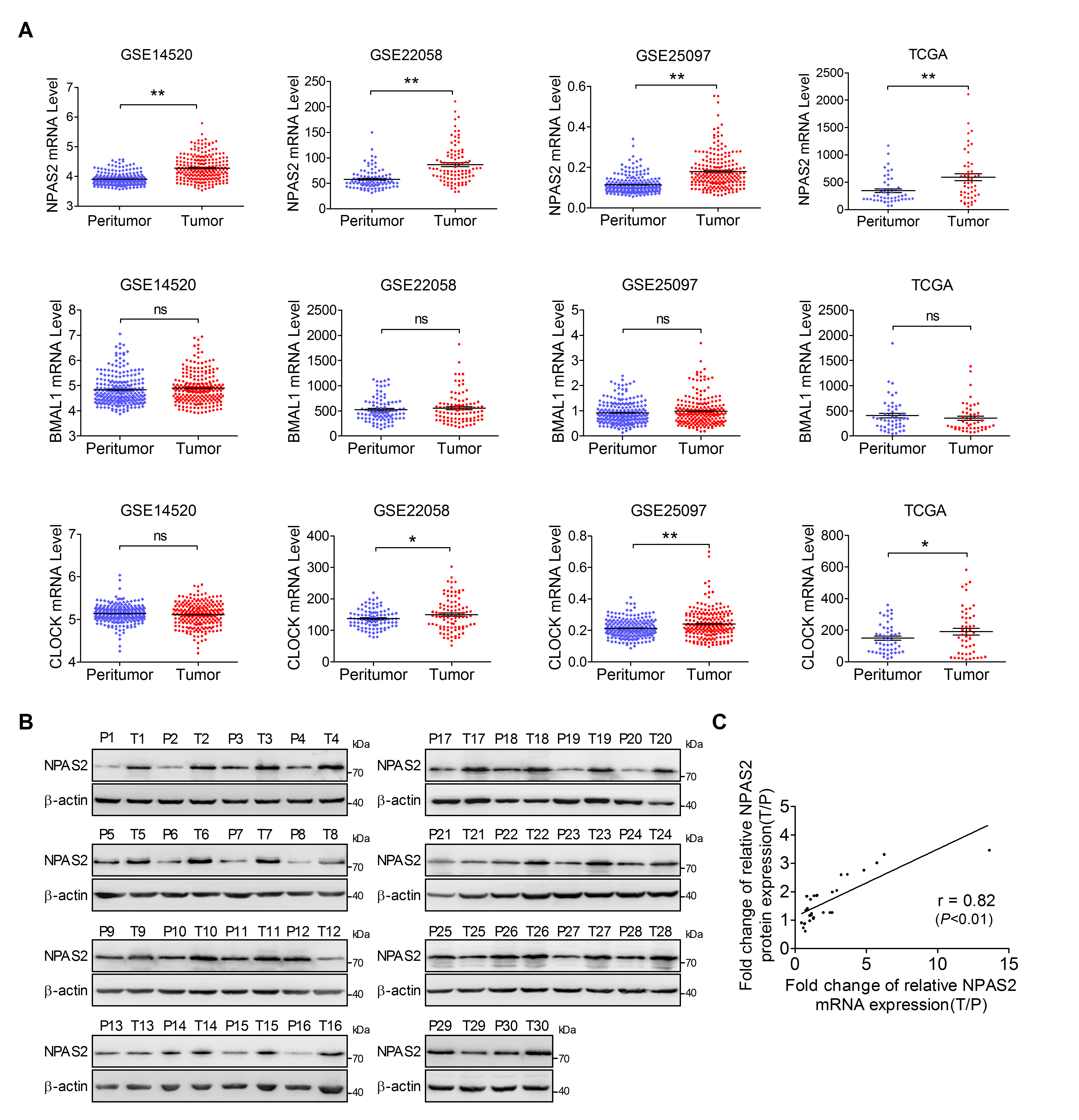
**

**Figure S1.** **Expression levels of NPAS2 in HCC patients.** (**A**) The public data of HCC were obtained from GEO and TCGA databases, and the mRNA expression levels of NPAS2, BMAL1 and CLOCK in HCC and peritumor were statistically analyzed (**P* < 0.05; ***P* < 0.01). (**B**) Western blot analyses for expression levels of NPAS2 in 30 paired tissues from HCC patients. T, tumor; P, peritumor. Experiments were repeated twice with independent protein extracts and representative data were presented. (**C**) Spearman correlation analysis between fold-change of relative protein expression (T/P) and mRNA expression (T/P) for NPAS2 was carry out in 30 paired HCC tissues.


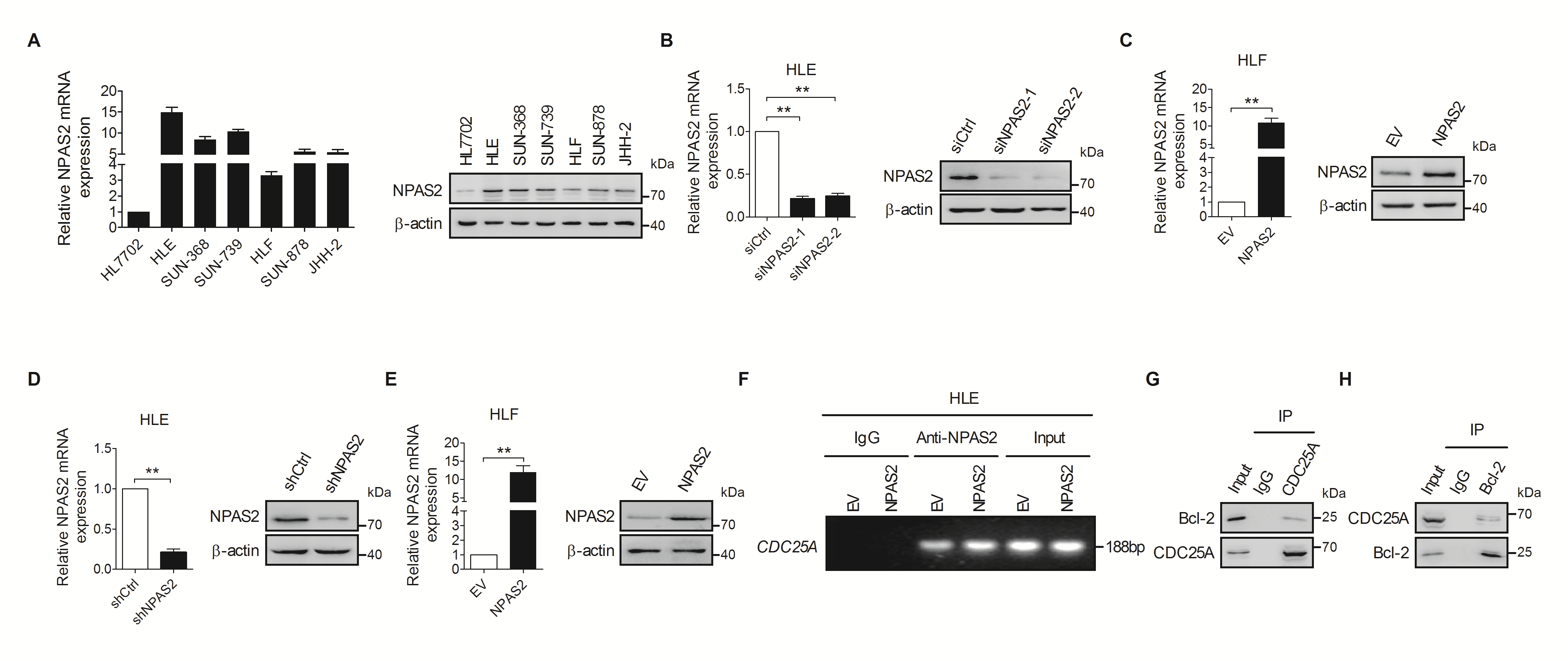


**Figure S2.** **Expression levels of NPAS2 in HLE and HLF cells.** (**A**) qRT–PCR and western blot analyses for NPAS2 expression were performed in a panel of HCC cell lines. (**B-C**) qRT–PCR and western blot analyses for NPAS2 expression were performed in HLE and HLF cells, which were transiently transfected with expression vector or siRNA as indicated. NPAS2, expression vector encoding NPAS2; EV, empty vector; siNPAS2-1 and siNPAS2-2, siRNAs against NPAS2; siCtrl, control siRNA. (**D-E**) qRT–PCR and western blot analyses for NPAS2 expression were performed in HLE cells stably expressing shRNA against NPAS2 (shNPAS2) and HLF cells stably expressing exogenous NPAS2 (NPAS2). shCtrl, control siRNA expression vector; EV, empty vector. (**F**) Amplification of the CDC25A promoter sequence from chromatin immunoprecipitation (ChIP) DNA was performed (HLE). Input and IgG served as positive and negative controls, respectively. The electrophoresis results of PCR products confirmed that NPAS2 binds to the CDC25A promoter. (**G** and **H**) Co-immunoprecipitation (Co-IP) assay using control IgG and anti-CDC25A or anti-Bcl-2 antibody was carried out using extracts prepared from HLE cells. The presence of CDC25A or Bcl-2 in these immunoprecipitates was evaluated by immunoblotting (WB). Experiments were repeated twice with independent cell extracts and representative data were presented. Data shown are the mean ± SEM from three independent experiments. *, *P* < 0.05; **, *P* < 0.01.

**
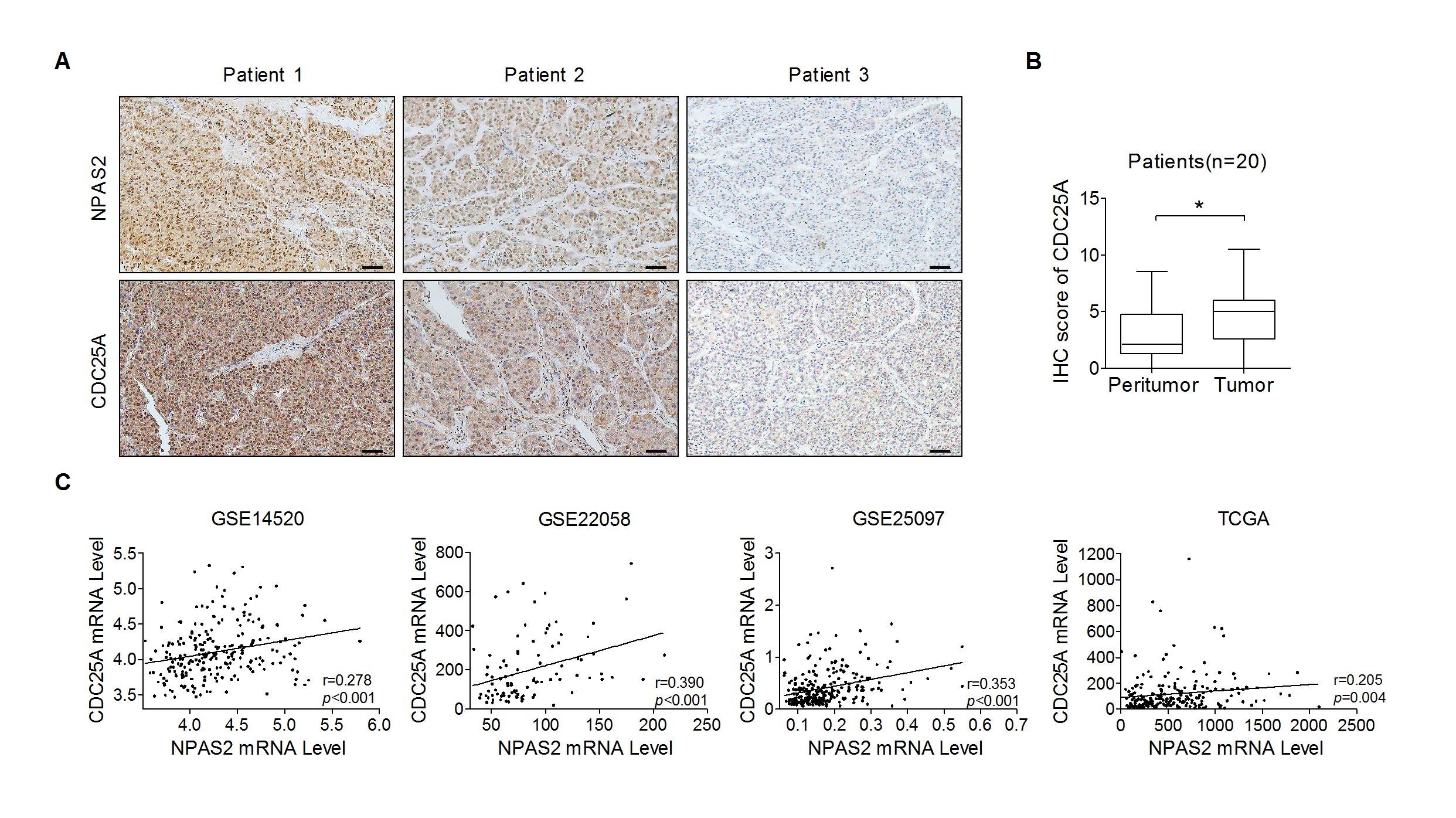
**

**Figure S3. Correlation analysis between NPAS2 and CDC25A.** (**A**) Representative immunohistochemical (IHC) staining images of NPAS2 and CDC25A in HCC tissues. Scale bar, 50μm. **(B)** Representative IHC scores of CDC25A in paired HCC tissues (n=20). *, *P* < 0.05; **, *P* < 0.01. (**C**) Correlation analysis between NPAS2 and CDC25A based on four public datasets of mRNA expression in HCC tissues, including RNA-seq data from TCGA and microarray data of GSE14520, GSE22058 and GSE25097 from GEO database.

**
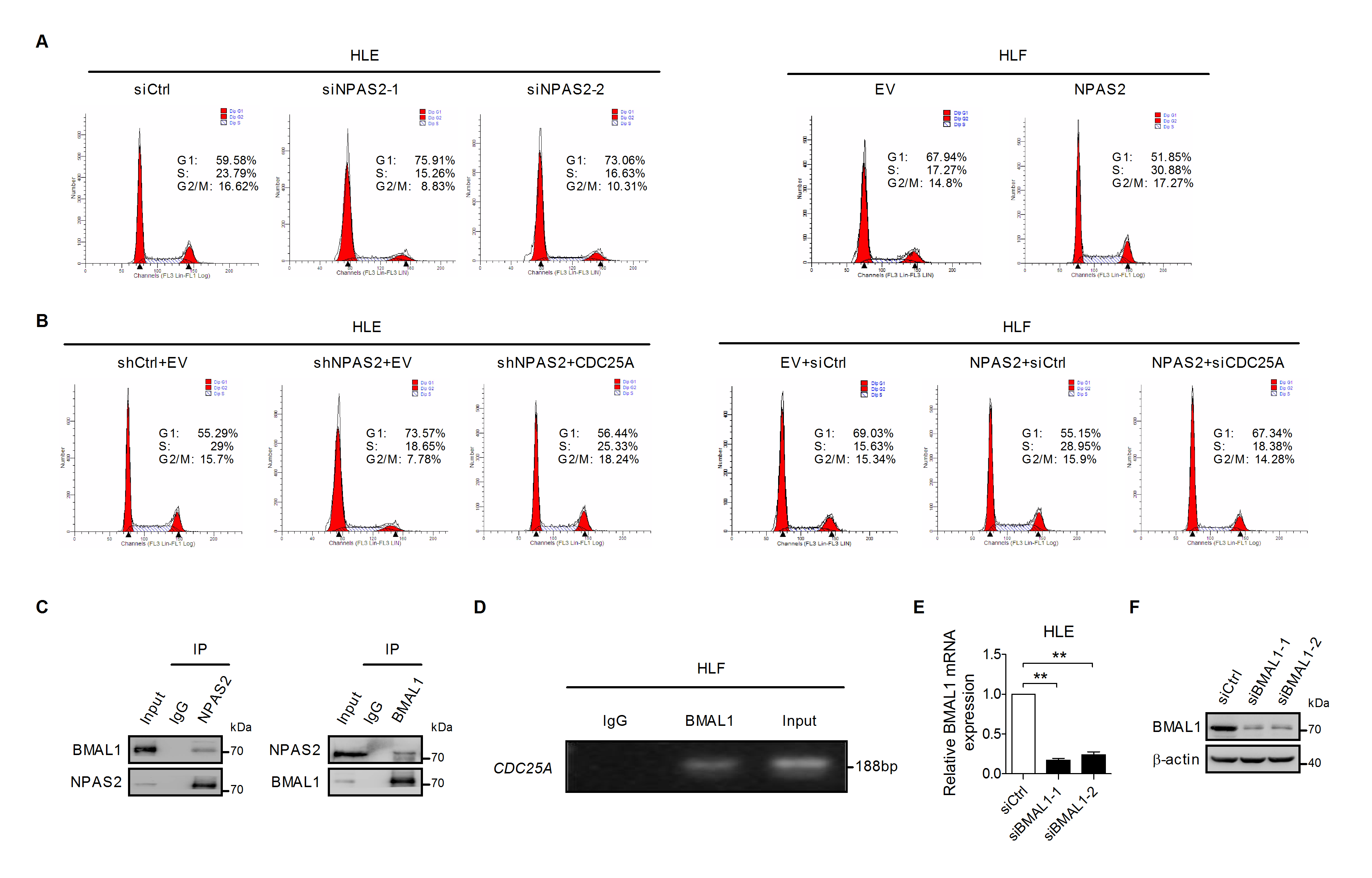
**

**Figure S4. NPAS2 in association with BMAL1 in HCC cells.** (**A**) Cell cycle analysis by flow cytometry in HLE and HLF cells after transiently transfected with siRNA or expression vector as indicated. (**B**) Cell cycle analysis by flow cytometry was performed in HLE and HLF cells with treatment as indicated. (**C**) Co-immunoprecipitation (Co-IP) assay using control IgG and anti-NPAS2 or anti-BMAL1 antibody was carried out using extracts prepared from HLF cells. The presence of BMAL1 or NPAS2 in these immunoprecipitates was evaluated by immunoblotting (WB). (**D**) Amplification of the CDC25A promoter sequence from ChIP DNA was performed (HLF). Input and IgG served as positive and negative controls, respectively. (**E** and **F**) qRT–PCR and western blot analyses for BMAL1 expression were performed in HLE cells, which were transiently transfected with siRNA as indicated. siBMAL1-1 and siBMAL1-2, siRNAs against BMAL1; siCtrl, control siRNA. Experiments were repeated twice with independent cell extracts and representative data were presented. Data shown are the mean ± SEM from three independent experiments. *, *P* < 0.05; **, *P* < 0.01.

**Supplementary materials and methods**

**Collection of tissue samples and clinical data**

Tissue samples from HCC patients were collected at Xijing Hospital affiliated with the Fourth Military Medical University in Xi’an, China. The eligibility criteria for HCC patient recruitment were set as follows: (1) histologically-confirmed hepatocellular carcinoma (HCC); (2) receiving surgical resection; (3) availability of complete clinical and follow-up data; (4) no preoperative anticancer treatment; (5) no history of other malignancy; and (6) alive at least 1 month after surgery. The demographic information, clinical and follow-up data of each patient were collected by well-trained staff interviewers or clinical specialists and summarized in Supplementary Table 1. The last follow-up date was February 2016 and the median follow-up duration was 30.3 months (ranging from 3.5 to 85 months). Overall survival was defined as the time from surgery to HCC-specific death or last follow-up. Recurrence-free survival (RFS) time was defined as the interval from the date of the surgery to the date of recurrence or last follow-up. The study was approved by the Ethics Committee of the Fourth Military Medical University and written informed consent was obtained from all participants.

**Knockdown, forced expression of target genes**

For generation of shRNA expression vectors, a small hairpin RNA (shRNA) containing specific sequences targeting the human NPAS2 mRNA sequence (5'-CGUCGGAUGUCAUGGAUCA-3') was cloned into the pSilencer™ 3.1-H1 puro vector (Ambion). A control shRNA (5'- UUCUCCGAACGUGUCACGUTT-3') was also cloned into the pSilencer™ 3.1-H1 puro vector, which was used as a silencing negative control. For overexpression, the coding sequences of NPAS2 and CDC25A were amplified from cDNA derived from HL7702 cell using primers listed in Supplementary Table 3 and cloned into the pcDNA^TM^3.1(+) vector (Invitrogen,V790-20). CDC25A(C431S) was generated by using the Q5 Site-Directed Mutagenesis Kit (NEB, E0552S) according to the manufacturer's instructions. The primers for generating CDC25A(C431S) were listed in the Supplementary Table 3. For transfection, HLF and HLE cells were seeded in 6-well plates to 60-80% confluence. Then the vectors were respectively transfected into HCC cells using the Lipofectamine 2000 reagent (Invitrogen, 11668019) according to the manufacturer’s protocol. Stable transfectants were selected using G418 sulfate (Sigma-Aldrich, A1720) for 3 weeks. All siRNAs were synthesized by GenePharma (Shanghai, China). The sequences of siRNA for NPAS2, CDC25A and BMAL1 are provided in Supplementary Table 3. Transfection was performed using lipofectamine 2000 (Invitrogen, 11668019), according to the manufacturer’s instructions.

**Quantitative real-time reverse transcription PCR (qRT-PCR)**

Total RNA was isolated using the Trizol Reagent (Invitrogen, 15596018). Complementary DNA (cDNA) was reversely transcribed using an PrimeScript RT Reagent kit with gDNA Eraser (Takara, RR047A) according to the manufacturer’s instructions. For the qRT-PCR analysis, SYBR Green PCR Kit (Takara, 639676) was used according to the manufacturer’s instructions. The relative expression level (defined as fold change) of the target gene was determined using a 2^−△△CT^ method. The expression level of the specific transcripts was normalized against that of β-actin. The primers for these transcripts were listed in the Supplementary Table 3. For mRNA expression level of target genes in HCC tissues, the fold change between tumor and adjacent non-tumor tissues was log_2_-transformed for further comparison.

**Western blot**

HCC tissues and cell lines were processed for western blot as previously described.(1) The band intensity on the western blots analyses was quantified using Quantity One software (Bio-Rad, Hercules, CA). The fold change between tumor and adjacent nontumor tissues were log_2_-transformed for further analysis. The primary antibodies used in this study and their working concentration were listed in Supplementary Table 4.

**H&E and immunohistochemistry staining**

H&E Staining was carried out following standard procedures. IHC staining and quantitative scoring analysis were performed as previously described.(2) Primary antibodies used in this study and their working concentration were listed in Supplementary Table 4.

**Cell viability and colony formation assay**

Cell viability was determined by the MTS assay (Promega Corporation, G3581) according to the manufacturer’s instructions. Briefly, HCC cells were plated in 96-well plates (Costar) at 1000 cells per well. After 12 h, cell viability and growth was measured by addition of 20 μl of MTS (0.2%)-PMS (0.092%; phenazine methosulfate, 20:1) solution and incubation for 2 h at 37°C. The microplates were read in a spectrophotometer at a wavelength of 490 nm. Each sample was analyzed in triplicate. To evaluate the colony formation ability, 1000 cells were seeded per well in 6-well plate and cultured for 14 days at 37°C in a humidified incubator with 5% carbon dioxide. After washing with PBS, colonies were fixed and stained with crystal violet and the number of colonies was counted.

**Ethynyl deoxyuridine (EdU) incorporation assays**

The proliferation ability of cells was analyzed using EdU incorporation assay kit (Ribobio, C10310-1) according to the manufacturer's instructions. Briefly, cells were incubated with 5 μM EdU in DMEM medium supplemented with 10% FBS for 2 h at 37°C. Cells were then washed with PBS, followed by fixation and permeabilization in PBS containing 4% formaldehyde, 0.5% tritonX-100 for 20 min. After extensive washing with PBS, cells were reacted with 1× Apollo® reaction cocktail (Ribobio, C10310-1) for 30 min. Subsequently, the DNA contents were stained with Hoechst 33342 for 30 min and visualized under a fluorescent microscope (DM5000B; Leica, Heerbrugg, Switzerland). The percentage of EdU-positive cells was determined.

**Cell apoptosis assays**

Cell apoptosis was determined with an Annexin V-FITC Apoptosis Detection Kit (BestBio, BB-4101-2) following the manufacturers’ instructions. Briefly, HCC cells seeded in 6-well plates were collected and resuspended with 500 μL binding buffer at a concentration of 10^6^ cells/mL. After adding 5 μL ANXA5-FITC and 5 μL PI, cells were mixed and incubated at room temperature in the dark for 15 min. The samples were analyzed with a flow cytometer (Beckman, Fullerton, CA). The terminal deoxynucleotidyl transferase–mediated dUTP nick-end labeling (TUNEL) assay was performed for apoptosis analysis in xenograft tissues as previously described.(3)

**Nude mice xenograft model**

Male BALB/c nude mice (4-6 weeks old) were randomly divided into groups. For the development of tumor xenografts in nude mice, 1x10^7^ HCC cells were injected into the flanks of male nude mice (n=7 in each group). Tumor length (L) and width (W) were measured using a vernier caliper every 3 days and tumor volume was calculated according to the formula (L x W^2^)/2 and presented as Mean ± SEM. Transplanted mice were sacrificed 24 days later, at which point the weight of tumors nodule were photographed and calculated. The study was approved by the ethics committee of the Fourth Military Medical University for animal research.

**Construction of reporter plasmids and site-directed mutagenesis**

Promoter sequences of CDC25A were obtained from UCSC Genome Browser. Then pGL3-Basic vectors (Promega, Madison, WI) inserted by truncated portions of CDC25A promoter was generally constructed by PCR amplification of selected regions with primers listed in Supplementary Table 3. This construct corresponds to the sequence from nt-2057 to nt+59 (relative to the transcriptional start site) of the 5’-flanking region of the human CDC25A gene. Site-directed mutagenesis was performed using the Q5 Site-Directed Mutagenesis Kit (NEB, E0552S) according to the manufacturer's instructions. The second E-box was mutated (underlined) to ACAGGA (wt, CAAGTG). The sequences of PCR products were confirmed by sequencing (Sangon, Shanghai, China).

**Luciferase assay**

4 μg of CDC25A promoter constructs were co-transfected with Renilla luciferase expressing control vector into 1×10^6^ HCC cells. Transfected cells were cultured for 48h. Cells were lysed and the luciferase activates were determined using the Dual Luciferase Reporter Assay kit (Promega, E1910) according to the manufacturer’s instructions. The relative light units were measured by a Luminoscan Ascent Microplate Luminometer (Thermo Scientific). The firefly luciferase activity corresponding to a specific promoter construct was normalized to renilla luciferase activity.

**ChIP assays**

The ChIP assays were carried out using a ChIP assay kit (Cell Signaling, #9005), according to the manufacturers' instructions. Briefly, the cells were cross-linked with 1% formaldehyde for 10 min and then disrupted in cell lysate buffer. The chromatin DNA was used micrococcal nuclease to shear DNA to average length between 150bp and 900bp as verified by agarose gel. After the samples were sonicated to break nuclear membrane. The supernatant was then collected. Subsequently, the chromatin was immunoprecipitated with anti-NPAS2 antibody (1:100, Abcam, ab157165), anti-BMAL1 antibody (1:50, NOVUS, NB100-2288) or the equal amounts of negative control Normal Rabbit IgG. Final DNA extractions were PCR amplified using primer pairs within the regulatory region of the CDC25A. The PCR reactions generate a 188bp production and the specific primers are listed in Supplementary Table 3.

**Cell cycle assays**

Cell Cycle was determined by PI staining (BestBio, shanghai, China) following the manufacturers’ instructions. Briefly, HCC cells seeded in 6-well plates were harvested and fixed in 70% ethanol and stored at 4°C overnight. Cells then were incubated with RNase at 37°C for 30 min, and stained with PI (1mg/mL) for 30 min. Cell cycle analysis was performed by using a flow cytometry (Beckman, Fullerton, CA). The percentage of cells in the G1, S, and G2/M phases of cell cycle was determined by their DNA content.

**Immunoprecipitation (IP) and** **Co-immunoprecipitation (Co-IP) assays**

For IP and Co-IP assays, cells were lysed in lysis buffer and incubated with 25 μL protein A beads (Santa Cruz) supplemented with anti-CDK4 antibody (1:500, Proteintech, 11026-1-AP), anti-CDC25A antibody (1:300, Proteintech, 55031-1-AP), anti-Bcl-2 antibody (1:500, Proteintech, 12789-1-AP), anti-NPAS2 antibody (1:50, Santa Cruz, sc-134404) and anti-BMAL1 antibody (1:300, Proteintech, 14268-1-AP) overnight at 4°C. After washed 3 times with cold lysis buffer, the immunoprecipitated samples or normalized amounts of total lysates were analyzed through immunoblotting (WB).

**Statistical analysis**

Experiments were repeated 3 times, where appropriate. Data representing mean ±standard error of the mean (SEM). Statistics were performed using SPSS 17.0 software (SPSS, Chicago, IL) and P < 0.05 was considered significant. Unpaired student t tests were used for comparisons between 2 groups where appropriate. Correlations between measured variables were tested by Spearman rank correlation analyses. For prognosis analysis, the IHC scores of NPAS2 were divided into high or low level by the median value for further analysis. The Kaplan-Meier survival curve and log-rank test were used to distinguish subgroup patients who had different overall survival.

**Supplementary Tables**

**Supplementary Table 1. Relationship between tumor NPAS2 expression and clinicopathologic features of HCC patients.**

| Variables | No. of cases | NPAS2 expression | | *P* value |
| --- | --- | --- | --- | --- |
|  |  | Low | High |  |
| All | 217 | 108 | 109 |  |
| Age |  |  |  | 0.730 |
| <55 | 101 | 49 | 52 |  |
| >=55 | 116 | 59 | 57 |  |
| Gender |  |  |  | 0.817 |
| Female | 27 | 14 | 13 |  |
| Male | 190 | 94 | 96 |  |
| HBsAg |  |  |  | 0.458 |
| Negative | 19 | 11 | 8 |  |
| Positive | 198 | 97 | 101 |  |
| AFP (ug/ml) |  |  |  | 0.024 |
| <200 | 116 | 66 | 50 |  |
| >=200 | 101 | 42 | 59 |  |
| Maximum diameter of lesion |  |  |  | 0.012 |
| <5 | 181 | 97 | 84 |  |
| >=5 | 36 | 11 | 25 |  |
| PVTT |  |  |  | 0.113 |
| No | 196 | 101 | 95 |  |
| Yes | 21 | 7 | 14 |  |
| TNM stage |  |  |  | 0.061 |
| I+ II | 176 | 93 | 83 |  |
| III+ IV | 41 | 15 | 26 |  |
| Differentiation grade |  |  |  | 0.359 |
| I+ II | 70 | 38 | 32 |  |
| III | 147 | 70 | 77 |  |
| Treatment |  |  |  | 0.556 |
| Hepatectomy | 163 | 83 | 80 |  |
| Hepatectomy + adjuvant TACE | 54 | 25 | 29 |  |

**Abbreviations**: HCC, hepatocellular carinoma; HBsAg, hepatitis B surface antigen; AFP, α -fetoprotein; PVTT, portal vein tumor thrombosis; TNM, tumor-nodes-metastases; TACE, transcatheter arterial chemoembolization; * P value < 0.05 was considered statistically significant.

**Supplementary Table 2. Public datasets used for bioinformatics analysis.**

| **Accession No.** | **Platform** | **Probes/**  **Genes** | **HCC Sample**  **No.** | **Patient Ethnicity** | **Etiology** | **Source URL** |
| --- | --- | --- | --- | --- | --- | --- |
| GSE14520 | Affymetrix Human Genome U133A 2.0 Array  Affymetrix HT Human Genome U133A Array | 22277/13518  22268/14181 | 247 | Chinese | HBV | http://www.ncbi.nlm.nih.gov/geo/query/acc.cgi?acc=GSE14520 |
| GSE22058 | Rosetta/Merck Human RSTA Affymetrix 1.0 | 43483/18503 | 96 | Chinese | HBV | http://www.ncbi.nlm.nih.gov/geo/query/acc.cgi?acc=GSE22058 |
| GSE25097 | Rosetta/Merck Human RSTA Affymetrix 1.0 | 37583/18076 | 268 | Chinese | HBV | http://www.ncbi.nlm.nih.gov/geo/query/acc.cgi?acc=GSE25097 |
| TCGA | Illumina HiSeq | 20531 | 201 | Asia/White /Black | HBV/  HCV | http://cancergenome.nih.gov/ |

**Supplementary Table 3. Sequences of primers and siRNAs.**

| **Primer name** | **Sequences** | **Enzyme Concentrationdilutions** |
| --- | --- | --- |
| **1. Primers for real-time PCR:** |  |  |
| *NPAS2* forward primer: | TCTGGATCACAGAGCACCTC |  |
| reverse primer: | CAGGAGCTCCAGGTCATCA |  |
| *β-actin* forward primer: | CCCAGCCATGTACGTTGCTA |  |
| reverse primer: | TCACCGGAGTCCATCACGAT |  |
| *CDC25A* forward primer: | GTGAAGGCGCTATTTGGCG |  |
| reverse primer: | TGGTTGCTCATAATCACTGCC |  |
| *ELF4* forward primer: | CATCATAACAGACGGGACCTTG |  |
| reverse primer: | GCTGGGAGACTCCATATTGAGTA |  |
| *CDKN2AIP* forward primer: | CTTCCTCGGGTGCCGATAC |  |
| reverse primer: | ACCCCTTCATTGCTACTCGAT |  |
| *POU4F2* forward primer: | CAAGCAGCGACGCATCAAG |  |
| reverse primer: | GGGTTTGAGCGCGATCATATT |  |
| *Bcl-2* forward primer: | GGTGGGGTCATGTGTGTGG |  |
| reverse primer: | CGGTTCAGGTACTCAGTCATCC |  |
| *BMAL1* forward primer: | CATTAAGAGGTGCCACCAATCC |  |
| reverse primer: | TCATTCTGGCTGTAGTTGAGGA |  |
| **2.Primers for gene cloning** | |  |
| NPAS2 forward primer: | GGGGTACCATGGATGAAGATGAGAAAGACAG | Kpn I |
| reverse primer: | GCTCTAGATTATCGGGGCGGCTGCTGGA | Xba I |
| CDC25A forward primer: | CCCAAGCTTATGGAACTGGGCCCGGAGC | HindIII |
| reverse primer: | CGGGGTACCTCAGAGCTTCTTCAGACGAC | Kpn I |
| CDC25A(C431S) forward primer: | TGAGCGAGTTTTCTTCTGAGAGAG |  |
| reverse primer: | GAGTGAGTGAAACACAACAATGAC |  |
| **3.Primers for CDC25A promoter construct** | |  |
| (-2057/+59)CDC25A forward primer: | CGCGGTACCCCTTCTCCAGCAACCTTGACC | Kpn I |
| (-1415/+59) CDC25A forward primer: | CGCGGTACCTACGACAGGGAGAACTATTTG | Kpn I |
| (-1247/+59) CDC25A forward primer: | CGCGGTACCGTCTGGTGTTGGGGCCAAAGG | Kpn I |
| (-1061/+59) CDC25A forward primer: | CGCGGTACCAGCCACTAAGCCCAGCAGTAG | Kpn I |
| (-597/+59) CDC25A forward primer: | CGCGGTACCGTCATTGCATTCCCTCCCTCA | Kpn I |
| reverse primer: | CCGCTCGAGTGTCTTCGCTGTTCTCCCACC | Xho I |
| **4.** **Primers for CDC25A promoter site-directed mutagenesis** | |  |
| (-1061/+59) CDC25A mutation forward primer: | GGAACAGTGTacAGgaCATAAGCATTATGTAAACTTTAG | |
| (-1061/+59) CDC25A mutation reverse primer: | CAGTGTGTTAATCTGTATTAATTC |  |
| **5. Primers used for ChIP in the CDC25A promoter** | |  |
| CDC25A forward primer: | TATGTCACAGAGTTGTAAAG |  |
| reverse primer: | GATGCTCTTAGAATCATTTC |  |
| **6. siRNAs** |  |  |
| NPAS2 siRNA 1 sense: | CGUCGGAUGUCAUGGAUCA |  |
| antisense: | UGAUCCAUGACAUCCGACG |  |
| NPAS2 siRNA 2 sense: | UCAAAGAGCUCAGUUCCAU |  |
| antisense: | AUGGAACUGAGCUCUUUGA |  |
| CDC25A siRNA sense: | GCUCUGAAGAGGAGCCAUU |  |
| antisense: | AAUGGCUCCUCUUCAGAGC |  |
| BMAL1 siRNA 1 sense: | GCCUUCAGUAAAGGUUGAA |  |
| antisense: | UUCAACCUUUACUGAAGGC |  |
| BMAL1 siRNA 2 sense: | CCUGCAUCCUAAAGAUAUU |  |
| antisense: | AAUAUCUUUAGGAUGCAGG |  |
| Control siRNA sense: | UUCUCCGAACGUGUCACGU |  |
| antisense: | ACGUGACACGUUCGGAGAA |  |

**Supplementary Table 4. Primary antibodies used for Western blotting and Immunohistochemistry analysis.**

| **Antibody** | **Company (Cat. No.)** | **Working dilutions diDIConcentrationdilutions** |
| --- | --- | --- |
| NPAS2 | NOVUS (NBP1-31363) | WB: 1/1000 IHC:1/200 |
| CDC25A | Proteintech (55031-1-AP) | WB: 1/1000 IHC:1/400 |
| Cytochrome c | Proteintech (10993-1-AP) | WB: 1/1000 |
| COX4I1 | ABGENT (#AP9153a) | WB: 1/1000 |
| Caspase-9 | Proteintech (66169-1-Ig) | WB: 1/1000 |
| Caspase-3 | Proteintech (25546-1-AP) | WB: 1/800 |
| PARP | Cell Signaling (#9532) | WB: 1/1000 |
| Ki67 | FuzhouMaixin(#MAB-0129) | IHC: 1/150 |
| β-actin | Beijing TDY (TDY051C) | WB: 1/3000 |
| CDK2 | Proteintech (10122-1-AP) | WB: 1/1000 |
| Phospho-Cdk(Thr14/Tyr15)-R | Santa Cruz (sc-28435-R) | WB: 1/100 |
| CDK4 | Proteintech (11026-1-AP) | WB: 1/500 |
| Phospho-Tyr | Millipore (05-321) | WB: 1/1000 |
| CDK6 | Proteintech (19117-1-AP) | WB: 1/1000 |
| Phospho-Cdk6(Tyr24) | Santa Cruz (sc-293097) | WB: 1/100 |
| Cyclin A2 | Proteintech (18202-1-AP) | WB: 1/1000 |
| Cyclin D1 | Cell Signaling (#2926) | WB: 1/2000 |
| Cyclin E1 | Cell Signaling (#4129) | WB: 1/1000 |
| Bax | Proteintech (50599-2-Ig) | WB: 1/800 |
| Bak | Proteintech (14673-1-AP) | WB: 1/800 |
| Bcl-2 | Proteintech (12789-1-AP) | WB: 1/1000 |
| Bcl-xl | Proteintech (10783-1-AP) | WB: 1/500 |
| Phospho-Bcl-2 (Thr69) | Thermo (PA5-36742) | WB: 1/1000 |
| BMAL1 | Proteintech(14268-1-AP) | WB: 1/1500 |

**Supplementary Table 5. PrognoScan database used for meta-analysis of the prognostic value of NPAS2.**

| **Dataset** | **Cancer Type** | **Endpoint** | **Probe ID** | **Sample No.** | **COX p-value** | **HR [95% CI]** |
| --- | --- | --- | --- | --- | --- | --- |
| GSE1456-GPL96 | Breast cancer | RFS | 213462_at | 159 | 0.0296 | 0.55 [0.32 - 0.94] |
| GSE1456-GPL96 | Breast cancer | DSS | 213462_at | 159 | 0.0465 | 0.52 [0.28 - 0.99] |
| GSE2034 | Breast cancer | DMFS | 205460_at | 286 | 0.0361 | 0.73 [0.54 - 0.98] |
| GSE3143 | Breast cancer | OS | 39548_at | 158 | 0.0400 | 0.53 [0.29 - 0.97] |
| GSE6532-GPL570 | Breast cancer | DMFS | 213462_at | 87 | 0.0316 | 0.59 [0.37 - 0.96] |
| GSE6532-GPL570 | Breast cancer | RFS | 213462_at | 87 | 0.0316 | 0.59 [0.37 - 0.96] |
| GSE6532-GPL570 | Breast cancer | DMFS | 39548_at | 87 | 0.0406 | 0.37 [0.14 - 0.96] |
| GSE6532-GPL570 | Breast cancer | RFS | 39548_at | 87 | 0.0406 | 0.37 [0.14 - 0.96] |
| GSE31210 | Lung cancer | RFS | 39548_at | 204 | 0.0264 | 2.16 [1.09 - 4.25] |
| GSE31210 | Lung cancer | RFS | 205460_at | 204 | 0.0294 | 1.33 [1.03 - 1.71] |
| GSE8894 | Lung cancer | RFS | 205459_s_at | 138 | 0.0112 | 2.26 [1.20 - 4.24] |
| GSE30929 | Soft tissue cancer | DRFS | 39549_at | 140 | 0.0023 | 2.93 [1.46 - 5.85] |
| GSE30929 | Soft tissue cancer | DRFS | 213462_at | 140 | 0.0085 | 1.93 [1.18 - 3.14] |
| GSE30929 | Soft tissue cancer | DRFS | 205459_s_at | 140 | 0.0290 | 2.72 [1.11 - 6.69] |
| GSE30929 | Soft tissue cancer | DRFS | 39548_at | 140 | 0.0410 | 3.22 [1.05 - 9.89] |
| GSE26712 | Ovarian cancer | OS | 205460_at | 185 | 0.0173 | 0.54 [0.32 - 0.90] |
| GSE26712 | Ovarian cancer | OS | 205460_at | 185 | 0.0221 | 0.58 [0.37 - 0.93] |
| GSE9891 | Ovarian cancer | OS | 205459_s_at | 278 | 0.0043 | 0.67 [0.50 - 0.88] |
| GSE9891 | Ovarian cancer | OS | 205460_at | 278 | 0.0076 | 0.74 [0.59 - 0.92] |
| GSE9891 | Ovarian cancer | OS | 39548_at | 278 | 0.0164 | 0.76 [0.60 - 0.95] |
| GSE4271-GPL96 | Brain cancer | OS | 39549_at | 77 | 0.0182 | 1.41 [1.06 - 1.89] |
| GSE4412-GPL96 | Brain cancer | OS | 39549_at | 74 | 0.0202 | 1.47 [1.06 - 2.04] |
| GSE4412-GPL96 | Brain cancer | OS | 213462_at | 74 | 0.0223 | 1.79 [1.09 - 2.96] |
| GSE4475 | Blood cancer | OS | 39549_at | 158 | 0.0166 | 0.22 [0.06 - 0.76] |
| GSE8970 | Blood cancer | OS | 205459_s_at | 34 | 0.0358 | 1.89 [1.04 - 3.44] |
| GSE19234 | Skin cancer | OS | 213462_at | 38 | 0.0030 | 2.21 [1.31 - 3.73] |
| GSE19234 | Skin cancer | OS | 39549_at | 38 | 0.0034 | 2.12 [1.28 - 3.52] |
| GSE19234 | Skin cancer | OS | 39548_at | 38 | 0.0056 | 3.02 [1.38 - 6.60] |
| GSE13507 | Bladder cancer | DSS | ILMN_1765558 | 165 | 0.0026 | 0.57 [0.40 - 0.82] |

**Abbreviations:** RSF, Relapse Free Survival; DMFS, Distant Metastasis Free Survival; OS, Overall Survival; DSS, Disease Specific Survival; DRFS, Distant Recurrence Free Survival; HR, hazard ratio; 95%CI, 95% confidential interval.

**Supplementary references**

1. Li J, Huang Q, Long X, Zhang J, Huang X, Aa J, Yang H, et al. CD147 reprograms fatty acid metabolism in hepatocellular carcinoma cells through Akt/mTOR/SREBP1c and P38/PPARalpha pathways. *J Hepatol* 2015;63:1378-1389.

2. Huang Q, Li J, Xing J, Li W, Li H, Ke X, Zhang J, et al. CD147 promotes reprogramming of glucose metabolism and cell proliferation in HCC cells by inhibiting the p53-dependent signaling pathway. *J Hepatol* 2014;61:859-866.

3. Huang Q, Zhan L, Cao H, Li J, Lyu Y, Guo X, Zhang J, et al. Increased mitochondrial fission promotes autophagy and hepatocellular carcinoma cell survival through the ROS-modulated coordinated regulation of the NFKB and TP53 pathways. *Autophagy* 2016;12:999-1014.
